# The Value of Cardiac Magnetic Resonance Imaging in Identification of Rare Diseases Mimicking Hypertrophic Cardiomyopathy

**DOI:** 10.3390/jcm10153339

**Published:** 2021-07-28

**Authors:** Tingting Fang, Jie Wang, Yu Kang, Fuyao Yang, Yuanwei Xu, Ke Wan, Jiayu Sun, Yuchi Han, Yucheng Chen

**Affiliations:** 1 Department of Cardiology, West China Hospital, Sichuan University, Chengdu 610041, China; ftt168mail@163.com (T.F.); WangJie1993@wchscu.cn (J.W.); kangyu_46@scu.edu.cn (Y.K.); yangfuyao2013@163.com (F.Y.); xuyuanwei_cardiac@163.com (Y.X.); wangke@wchscu.cn (K.W.); 2 Department of Radiology, West China Hospital, Sichuan University, Chengdu 610041, China; sjy080512@163.com; 3 Department of Medicine (Cardiovascular Division), University of Pennsylvania, Philadelphia, PA 19104, USA; 4 Center of Rare Diseases, West China Hospital, Sichuan University, Chengdu 610041, China

**Keywords:** glycogen storage disease, late gadolinium enhancement (LGE), T1 mapping, hypertrophic cardiomyopathy (HCM)

## Abstract

Background: The cardiac Magnetic Resonance Imaging (MRI) characteristics of rare diseases with the hypertrophic cardiomyopathy (HCM) phenotype are not well defined. Methods: Seventy-three sequential patients and 34 of their relatives, who have the HCM phenotype, were included. All subjects underwent cardiac MRI and genetic testing. Results: Of these 107 patients with phenotypic HCM, seven rare diseases were identified: four cases with LAMP2, one case with PRKAG2, one case with TTR mutation, and one case with senile systemic amyloidosis. Subjects with rare diseases had diffuse LGE, and the percentage of those with LGE was significantly higher than that of other HCM (median: 18.9%, interquartile range (IQR): 14.05 to 28.2% versus 7.8%, IQR: 4.41 to 14.56%; *p* = 0.003). Additionally, global T1 and ECV were significantly higher in subjects with rare diseases (global T1: 1423.1 ± 93.3 ms versus 1296.2 ± 66.6 ms; global ECV: 44.3 ± 11.5% versus 29.9 ± 4.5%; all *p* < 0.001). Conclusions: Cardiac MRI suggests the existence of distinct imaging characteristics, including via LGE and T1 mapping, among rare diseases that mimic HCM and HCM itself.

## 1. Introduction

Hypertrophic cardiomyopathy (HCM) is the most common primary cardiomyopathy and manifests as unexplained myocardial hypertrophy. The prevalence of HCM is 1:500 in the general adult population [1]. HCM is an autosomal dominant genetic disease with 40–60% of patients identified as having mutations of sarcomere-related genes. The etiology in 5–10% of the HCM phenotype is heterogeneous, including metabolic disease, infiltrative disease, and neuromuscular disease [2]. In general, most HCM has a benign prognosis. However, some rare diseases with the HCM phenotype have a malignant prognosis if left untreated [3]; thus, it is imperative to identify these rare diseases in patients with phenotypic HCM in clinical practice. In previous studies, a group of heterogeneous diseases with specific genetic mutations was found to have similar HCM phenotypes, such as Anderson–Fabry disease (GLA), PRKAG2 syndrome, Danon disease (LAMP2), Pompe’s disease (GAA), LEOPARD, Noonan’s syndromes (RAF1 and PTPN11), TTR (amyloidosis), and Friederich’s ataxia (FXN) [4,5]. However, the prevalence of these rare diseases is diverse in different regions [6]. At present, it remains a challenge to accurately identify these rare diseases among patients with phenotypic HCM. Currently, genetic testing and myocardial biopsy are gold standards in the diagnosis of these rare diseases. However, difficulties remain in choosing which patients to refer for whole genome genetic testing and myocardial biopsy among patients with phenotypic HCM due to the low prevalence of such rare diseases. Therefore, it is important to find non-invasive parameters to act as gatekeepers for further investigation of rare diseases in phenotypic HCM.

Cardiac Magnetic Resonance Imaging (MRI) offers accurate morphological, functional, and myocardial tissue characterization using late gadolinium enhancement (LGE) and T1/T2 mapping, and thus improves diagnosis in patients with HCM [7,8,9,10,11,12,13]. Compared with cardiac MRI, echocardiography and computerized tomography (CT) have certain technical limitations in the evaluation of rare diseases with hypertrophic phenotypes [14,15]. Regarding echocardiography, the required acoustic windows and inability of the technique to evaluate the myocardial histological characteristics decreases its sensitivity in detecting LV hypertrophy, whereas CT has radiation risks and is not conducive to the follow-up of these patients. However, multiple sequences of cardiac MRI, such as LGE and T1 mapping, can help to improve diagnosis and risk stratification [16,17,18,19,20]. LGE characteristics have been demonstrated to differentiate different etiologies of left ventricular hypertrophy [16,17], and T1 mapping can add additional values [18,19,20]. At present, scarce data exists regarding the exploration of the characteristics of rare diseases by cardiac MRI in patients presenting as phenotypic HCM. We hypothesize that cardiac MRI with comprehensive tissue characterization may be an effective imaging tool in discriminating rare etiologies of phenotypic HCM.

## 2. Materials and Methods

### 2.1. Subjects and Design of the Study

The study was a prospective observational trial. The study population was prospectively recruited from our clinical CMR registry, and was registered with the Chinese clinical trial registry (URL: http://www.chictr.org.cn, accessed on 25 June 2019; Unique identifier: ChiCTR1900024094). The study population consisted of 73 sequential patients with the HCM phenotype, and 34 relatives of these 73 patients who also have the HCM phenotype, who were referred for 3.0-T cardiac MR imaging from September 2016 to June 2017. The HCM phenotype was based on the presence of LV wall thickness ≥ 15 mm in one or more myocardial segments (or ≥13 mm in a first-degree relative of an index patient with HCM) measured by any imaging criteria, including echocardiography, CT scan, and cardiac MRI in the absence of secondary causes of hypertrophy [21]. All patients underwent cardiac MRI scans at our center. This study was approved by our Institutional Ethics Committee (2016 (355)), and written informed consent was obtained from each participant.

### 2.2. Genetic Testing and Data Analysis

We performed genetic testing in all subjects by next-generation sequencing (NGS) with a panel including 117 cardiomyopathy-related genes [22,23] and *TTR* gene (Table S1). Methods for genetic sequencing and in silico analysis were previously described [23]. Exon-enriched DNA was sequenced by the Illumina hiseq 2500 platform, following the manufacturer’s instructions (Illumina). Raw image files were processed using the BclToFastq (Illumina) for base calling and raw data generation. The low-quality variations were filtered using the quality score ≥ 20 (Q20). The sequencing reads were aligned to the NCBI human reference genome (hg19) using BWA. VarScan and GATK were used to analyze SNP (Single Nucleotide Polymorphism) and indel of the sequence. Data were analyzed as follows: (1) Synonymous changes and SNPs that had a minor allele frequency (MAF) higher than 5% were removed; (2) Nonsynonymous changes were filtered using SIFT software; (3) The relationship between the function of mutated genes and related diseases was analyzed. Genetic results were subsequently confirmed using the Sanger sequencing method. In addition, pathogenicity determination of gene mutation was performed following the American College of Medical Genetics and Genomics recommendations (ACMG) [24]. A patient was considered genotype positive when the mutation was classified to be likely pathogenic or pathogenic (classes IV and V). Variants were also considered pathogenic if published as causative HCM mutations in at least two independent peer-reviewed studies.

### 2.3. Cardiac MRI Imaging

ECG gated cardiac MRI imaging was performed on a 3T scanner (MAGNETOM Trio Tim, Siemens Healthcare Ltd., Erlangen, Germany) with a 32-channel cardiac phase-array receiver coil. Steady-state free precession (SSFP) cine images of the entire LV from the base to the apex in consecutive short-axis views were acquired during breath-holds, with the following parameters: repetition time, 3.4 ms; echo time, 1.3 ms; temporal resolution, 42 ms; flip angle, 50 degrees; field of view, 320–340 mm; matrix size, 256 × 144. The reconstructed in-plane spatial resolution was 1.4 × 1.3 mm and the slice thickness was 8 mm with no gap. Native T1 mapping was acquired before injection of gadolinium by motion-corrected modified Look-Locker inversion recovery (MOLLI) sequence with a 5(3)3 acquisition scheme. Parameters for MOLLI were as follows: TR 740 ms; TE 1.06 ms; FA 35°; bandwidth 930 Hz/pixel; initial T1 100 ms, with 80 ms increments; parallel imaging factor 2; matrix size 192 × 144; in-plane spatial resolution of 2.4 × 1.8 mm; and total acquisition time of 11 heartbeats. T1 mapping was repeated in the identical short-axis slices at 15 min after administration of gadolinium with a MOLLI sequence scheme 4(1)3(1)2. Hematocrit values were tested within 12 h of cardiac MRI examination. LGE images were acquired at 10–15 min after intravenous administration of 0.15 mmol/kg of gadopentetate dimeglumine (Magnevist, Bayer Schering Pharma, Berlin, Germany), using a phase-sensitive inversion recovery (PSIR) sequence with short-axis views (TR, 700 ms; TE, 1.56 ms; FA, 20°; matrix size, 256 × 144). The inversion time (TI) was individually optimized to a null normal myocardial signal using a TI scout sequence.

### 2.4. Cardiac MRI Analyses

All functional analysis, LGE quantitation, and T1 measurement were performed using commercially available software (Qmass 8.1; Medis Medical Imaging Systems, Leiden, The Netherlands). The tracing method of ventricular function and mass was in accordance with previous studies [25,26]. Maximal LV wall thickness was defined as the greatest dimension anywhere within the LV myocardium. LGE was interpreted by two experienced cardiac MRI readers. If there was a discrepancy, the third cardiac MRI expert would make the final decision. If LGE existence was determined, the pattern of LGE was first classified as infarction or non-infarction pattern, and further classified as subtype pattern (typical HCM pattern, amyloid-like pattern, Fabry-like pattern, or unclassified pattern). The LGE extent was quantitated in accordance with previous studies [27,28]. LGE was defined automatically by the myocardial signal intensity of 6 standard deviations (SD) from the normal myocardium. The total extent of LGE was calculated by summing LGE volume in each slice as a proportion of total LV myocardium (%LGE).

Myocardial T1 values before and after contrast were acquired on the motion-corrected images based on LV short-axis slices. The endocardial and epicardial contours were manually traced on the pre-and post-contrast T1-mapping images, using Qmass 8.1 software. Average myocardial T1 was obtained for each imaging slice. At the same time, blood T1 was obtained by locating a region of interest in the blood pool within the LV cavity in the pre-and post-contrast T1 mapping images, respectively. The ECV was calculated as follows: ECV = (1 − HCT) × ([1/T1myo post − 1/T1myo pre]/[1/T1blood post − 1/T1blood pre]) [29], and global native T1 and global ECV were calculated as the averages of basal, middle, and apical slices.

### 2.5. Statistical Analysis

Statistical analysis was performed using SPSS (version 22.0; SPSS Inc., Chicago, IL, USA) and MedCalc (version 13.0; Ostend, Belgium) software. The Shapiro–Wilk test was first used to test normality, and the unpaired T-test or Mann–Whitney U test was used when appropriate. Continuous variables are expressed as mean ± SD, and median or interquartile range (IQR) when appropriate. Categorical variables are expressed as N (%). Receiver operating characteristic (ROC) curve analysis was used to evaluate the performance of LGE and T1 mapping, and the area under the curve (AUC) was compared using a methodology of Delong et al. *p* < 0.05 was considered statistically significant.

## 3. Results

### 3.1. Demographic, Clinical, and Genotyping Data

After the 107 patients with phenotypic HCM underwent clinical evaluation and genetic testing, seven rare diseases were identified (four cases with *LAMP2*, one case with *PRKAG2*, one with *TTR* mutation, and one case with senile systemic amyloidosis). Genetic defects causing Pompe’s disease (GAA) and Fabry’s disease (*GLA*) were not found in this group of patients. In the remaining 100 patients, a pathogenic mutation coding sarcomere-protein was identified in 87 patients, including one MYH7 gene mutation in 47 patients and one MYBPC3 mutation in 20 patients. The remaining 13 patients were considered to be HCM with a negative genotype. Clinical, genetic, electrocardiogram (ECG), and cardiac MRI features in patients with glycogen storage disease and ATTR amyloidosis are presented in Table 1.

### 3.2. Comparison between HCM Patients and Rare Disease Patients Presenting as HCM Phenotype

The baseline clinical and cardiac MRI characteristics of the two groups are shown in Table 2. There were no significant differences in age, sex, systolic blood pressure (BP), diastolic BP, and LV end diastolic volume index (LVEDVi). Although maximal wall thickness was similar between the two groups (21.9 ± 6.0 mm (HCM) versus 22.1 ± 9.9 mm (rare diseases); *p* = 0.734), LVmassi trended higher in the rare disease group than in the HCM patient group (127.8 ± 94.4 g/m^2^ versus 67.1 ± 33.3 g/m^2^; *p* = 0.067). In addition, the rare disease group had significantly lower LVEF (43.8 ± 22.6% versus 60.5 ± 9.2%, *p* = 0.033) and RVEF (50.0 ± 12.3 versus 59.5 ± 10.3, *p* = 0.028).

### 3.3. “Non-Typical” HCM Pattern of LGE and Quantitation of LGE Extent in Rare Disease Patients

**Figure 1:**
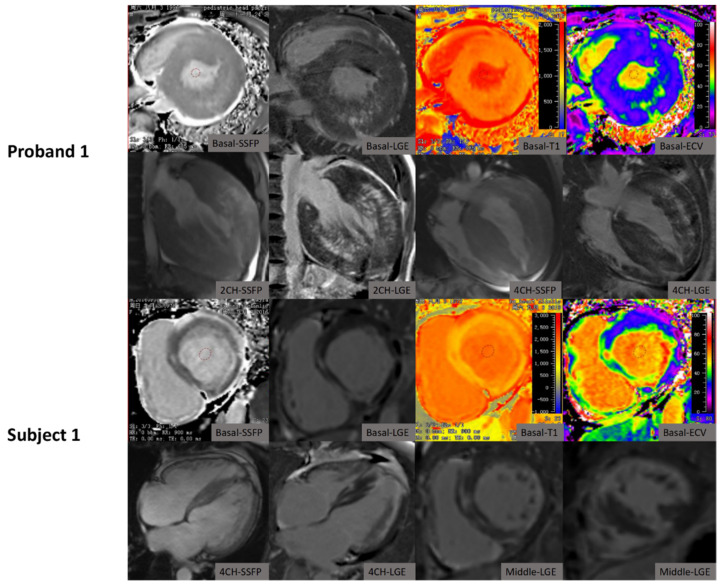
The characteristics of cardiac MRI in the proband 1 patient and the patient’s mother (Subject 1) with Danon disease.

**Figure 2:**
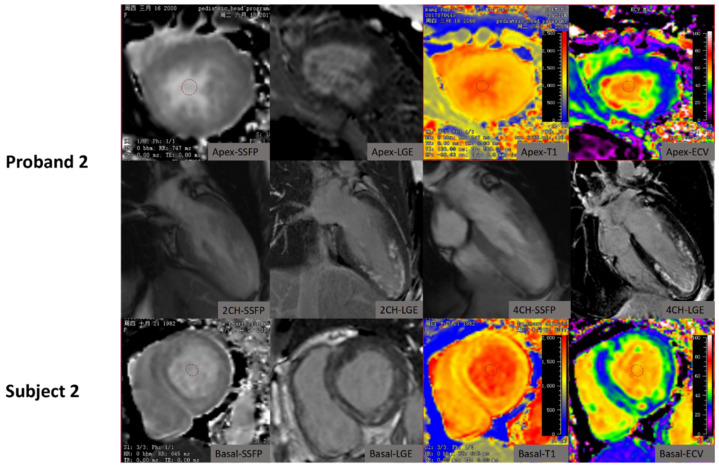
The characteristics of cardiac MRI in the proband 2 patient and the patient’s mother (Subject 2) with Danon disease.

**Figure 3:**
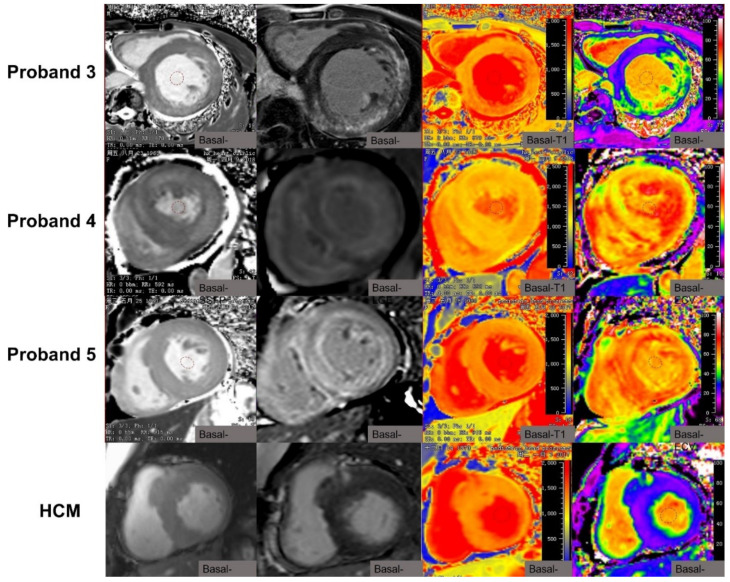
The characteristics of cardiac MRI in the proband 3 patient with *PSKAG2* syndrome, the proband 4 patient with senile systemic amyloidosis, and the proband 5 patient with *TTR*, and a HCM patient with the typical HCM LGE pattern.

The LGE patterns in rare disease patients are demonstrated in Figure 1, Figure 2 and Figure 3 and Table 1. The proband 1 patient with Danon disease showed a non-typical HCM LGE pattern with extensive fibrosis mainly in the mid-myocardium of the lateral wall with septal sparing (Figure 1), whereas the patient’s mother showed a heterogenous LGE pattern including non-ischemic sub-endocardial LGE, mainly in the lateral wall, and a transmural LGE pattern (Figure 1). In addition, a non-typical HCM LGE pattern with patchy circumferential non-ischemic sub-endocardial LGE was also identified in the proband 2 patient, which was consistent with our previous publication (Figure 2) [30]. Furthermore, the patient’s mother showed multiple focal LGE areas in the whole wall (Figure 2).

**Figure 4:**
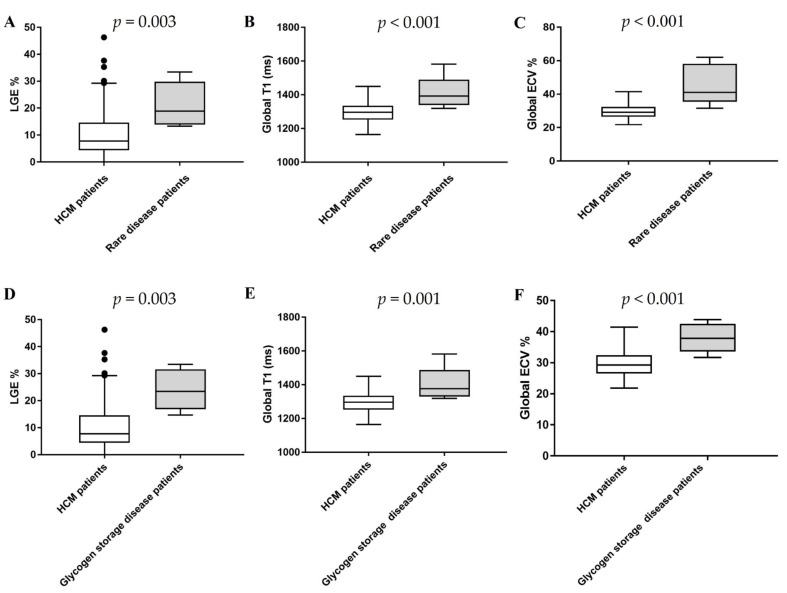
LGE, global native T1, and ECV comparisons between HCM patients and rare disease patients. The comparisons of LGE%, global T1 and ECV between the patients with rare diseases including patients with amyloidosis and HCM patients (**A**–**C**), The comparisons of LGE%, global T1 and ECV between the patients with rare diseases excluding patients with amyloidosis and HCM patients (**D**–**F**).

The proband 3 patient with *PSKAG2* syndrome showed a non-typical HCM LGE pattern with extensive mid-myocardial LGE mainly in the lateral wall (Figure 3). The proband 4 patient with senile systemic amyloidosis and the proband 5 patient with *TTR* showed non-typical HCM pattern with transmural LGE (Figure 3). The typical HCM LGE pattern with focal fibrosis in the septum between the right ventricular insertion points was also seen and is shown in Figure 3. The patients with rare diseases including patients with amyloidosis had a significantly higher extent of LGE than HCM patients (median: 18.9%, interquartile range (IQR): 14.05 to 28.2% versus 7.8%, IQR: 4.41 to 14.56%; *p* = 0.003, Figure 4), where LGE extent is quantified as %LGE. The patients with rare diseases excluding patients with amyloidosis had significantly higher LGE extent than HCM patients (median: 23.4%, IQR: 17.85 to 30.7% versus 7.76%, IQR: 4.41 to 14.56%; *p* = 0.003, Figure 4).

### 3.4. Native T1 and ECV by T1 Mapping

Similarly, the characteristics of T1 mapping in rare disease patients are demonstrated in Figure 1, Figure 2, Figure 3 and Figure 4 and Table 1. Native T1 and ECV were statistically different on the basal, middle, and apical slices in the two subgroups (Table 2). Rare disease patients including patients with amyloidosis had higher global native T1 and ECV than HCM patients (global T1: 1423.1 ± 93.3 ms (rare diseases) versus 1296.2 ± 66.6 ms (HCM); global ECV: 44.3 ± 11.5% (rare diseases) versus 29.9 ± 4.5% (HCM); all *p* < 0.001, Figure 4). In addition, rare disease patients excluding patients with amyloidosis had higher global native T1 and ECV than HCM patients (global T1: 1401.5 ± 104.63 ms (rare diseases) versus 1296.2 ± 66.57 ms (HCM), *p* = 0.001; global ECV: 37.98 ± 4.77% (rare diseases) versus 29.9 ± 4.5% (HCM); all *p* < 0.001, Figure 4).

## 4. Discussion

The present study presents the clinical and cardiac MRI characteristics of rare diseases with phenotypic HCM and HCM. Our study demonstrated that cardiac MRI, particularly using tissue characterization with extensive and “non-typical” HCM patterns of LGE, and significantly increased native T1 and ECV may provide clues to identifying rare diseases mimicking HCM phenotypes.

The European Heart Rhythm Association and the Heart Rhythm Society recommend clinical genetic testing for all HCM patients [31]. The European Society of Cardiology recommends genetic testing for patients with HCM who fulfill diagnostic criteria to enable cascade screening of their relatives (Class I) [2]. However, these guidelines do not specify the type of genetic testing to employ. In recent years, with the rapid development of the NGS method, analysis of gigabases of sequence information in a single run and unselective analysis of whole genomic DNA has become possible [32]. Additionally, the high efficiency and accuracy of the NGS method were verified in cardiomyopathies [33,34]. A well-planned panel and high-throughput NGS made it possible to screen most genetic variations associated with cardiomyopathy in all patients with HCM phenotypes. Using this method, we detected the rare gene mutation-related glycogen storage disease and *TTR* infiltrative disease.

To date, there have been few reports on cardiac MRI characteristics of Danon disease [30,35,36,37]. Dara et al. and Kownacka et al. reported LGE to be present in the anterior, lateral, and/or posterior walls, and one or both right ventricular insertion points, in three male patients, rather than at the septum between the right ventricular insertion points as in most cases of HCM [35,36,38]. Our report is consistent with these previous studies, and indicates that the “non-typical HCM” LGE pattern with septal sparing in male Danon patients should prompt genetic screening for Danon disease. In addition, in female Danon patients, there were heterogeneous LGE patterns. Dara et al. reported LGE presence in the lateral wall and superior and inferior RV insertion points, but not in the septum [35]. Vago et al. reported a patchy LGE pattern in the mid-myocardium [37]. In addition to these reports, the current study found non-ischemic sub-endocardial LGE, mainly in the anterior and lateral wall, and a patchy circumferential sub-endocardial LGE pattern in the mid-to-apical slice. Therefore, one may also need to consider Danon’s disease if there was a patchy non-ischemic sub-endocardial LGE pattern for female patients presenting with HCM. In addition, several case reports have described the characteristics of cardiac MRI-phenotypes in patients carrying the *PRKAG2* gene mutation [39,40,41,42]. Global sub-endocardial hyper-enhancement is a common LGE pattern in *PRKAG2* cardiomyopathy. Yogasundaram et al. and Yang et al. reported sub-endocardial or mid-myocardial LGE patterns in these patients [41,42]. Our report also confirms the mid-myocardial LGE pattern in *PRKAG2* cardiomyopathy. Concentric hypertrophy is the most common pattern in metabolic and infiltrative disorders. Global transmural LGE is the most frequent observation in cardiac ATTR amyloidosis [13,43]. Our report confirms transmural LGE patterns in two amyloidosis patients.

Previous studies showed that LGE allows for the identification of areas of focal fibrosis, and the T1 mapping technique can characterize diffuse changes in the myocardium [10]. In addition, native T1 and ECV have been shown to be elevated in HCM patients compared to healthy controls [44,45]. This increase can also be detected in mutation carriers with negative phenotypes [19]. Furthermore, Naharro A et al. found native T1 and ECV were elevated in ATTR compared with HCM (*p* < 0.001) and were both associated with a high diagnostic accuracy ((AUC): 0.87; 95% confidence interval (CI): 0.82 to 0.91) for T1 and an AUC of 0.91 (95% CI: 0.87 to 0.94) for ECV [20]. However, currently, data comparing tissue characteristics of Danon and *PRKAG2* diseases by LGE and T1 mapping with HCM patients are scarce. Our study found significant differences in LGE and T1 mapping between HCM patients and rare disease patients with HCM phenotypes. Further studies are needed to explore whether LGE% and global native T1 mapping can serve as a gatekeeper for further invasive myocardial biopsy or expensive genetic testing to distinguish between rare diseases and HCM patients.

Fabry disease (*GLA*) is a rare X-linked disorder of lysosomal metabolism and can also manifest as LV hypertrophy [46]. Previous studies have reported the decreased T1 indices in Fabry’s disease, compared with healthy volunteers or other diseases including HCM, hypertension, severe aortic stenosis, and cardiac amyloidosis [47]. Furthermore, Karur GR et al. confirmed that septal LV native T1 with a cutoff point of 1220 ms can be used to differentiate Fabry’s disease from HCM with a sensitivity of 97% and a specificity of 93% [18]. We did not find Fabry’s disease in the group of patients examined in the current study, and Fabry’s disease should be separated from the high T1 rare diseases because it has a lower T1 value.

The prognosis is poor in glycogen storage disease and cardiac amyloidosis if left untreated, underscoring the importance of identifying these patients presenting as HCM. Reliable imaging markers are crucial in differentiating rare diseases presenting as HCM from HCM itself, to allow further investigation to be triggered. Our study showed that quantitative analysis of LGE, native T1, and ECV may have the ability to detect these rare diseases in hypertrophic patients.

There are limitations to our study. First, a small number of rare diseases was found in this HCM phenotype cohort, which did not include all rare diseases, and the prevalence of these rare diseases varies by region. Therefore, our results need validation in another cohort of HCM patients.

In conclusion, our study showed that rare diseases among phenotypic HCM present with different tissue characteristics and can be differentiated from HCM. Cardiac MRI may serve as a gatekeeper for further whole genome testing or invasive myocardial biopsy.

## 5. Conclusions

This study demonstrated the value of cardiac MRI imaging characteristics in the distinction between rare diseases that mimic HCM and HCM itself.

## Figures and Tables

**Table 1:** Demographic, clinical, electrocardiogram and cardiac MRI-phenotypic characteristics of the rare disease patients with HCM phenotype.

Subjects	Genotype	Age (Year)	Sex	BMI (kg/m^2^)	Symptoms	NYHA Functional Classes	Syncope History	ECG	LV EF (%)	RV EF (%)	Max LVT (mm)	LGE (%)	LGE Pattern	Global Native T1 (ms)	Global ECV (%)	Clinical Course
Family 1																
Proband 1	*LAMP2* (c.64 + 1G > A)	20	M	18.0	Palpitation, DOE	II	Yes	NSR; ventricular preexcitation; NSST-T	59.8	50.4	42	33.4	Extensive fibrosis mainly in mid-myocardium of lateral wall with septal sparing	1376.5	35.5	ICD implantation; awaiting heart transplantation
Subject 1	*LAMP2* (c.64 + 1G > A)	44	F	23.4	DOE; edema	II	No	NSR; Short PR intervals; PVC	16.9	27.8	16	18.9	Non-ischemic sub-endocardial LGE mainly in lateral wall and transmural LGE pattern	1581.2	43.9	Heart failure managed; awaiting heart transplantation
Family 2																
Proband 2	*LAMP2* (c.973delC, p.L325Wfs*21)	17	F	20.0	Syncope,	I	Yes	NSR;ST-T	65.7	69.4	15	14.7	Patchy circumferential non-ischemic sub-endocardial LGE	1392.1	31.6	ICD implantation recommendation
Subject 2	*LAMP2* (c.973delC, p.L325Wfs*21)	42	F	18.8	DOE	II	No	NSR; NSST-T	64.0	55.9	14	23.4	Multiple focal LGE area in whole wall	1318.7	37.8	Heart failure managed
Proband 3	*PRKAG2* (c.166G > A, p.G56S;c.298G > A, p.G100S)	27	M	20.3	DOE;	II	Yes	NSR; Intra-ventricular conduction, signs of RVH and NSST-T	10.4	48.7	17	29.8	Extensive mid-myocardial LGE mainly in lateral wall	1339.0	41.1	ICD implantation; Awaiting heart transplantation; Died of progressive heart failure
Proband 4	Negative	55	F	18.4	DOE	II	Yes	LBBB	39.3	47.3	25	13.8	transmural LGE	1488.9	58.1	Awaiting heart transplantation
Proband 5	*TTR*(c.220G > C, p.E74Q)	58	F	17.6	DOE	II	Yes	NSR;NSST-T	50.8	50.8	19	13.3	transmural LGE	1465.5	62.0	Awaiting heart transplantation

M = male; F = female; ECG = electrocardiograph; DOE = dyspnea on exertion; NSST-T = non-specific ST-T changes; NSR = normal sinus rhythm; ICD = implantable cardioverter-defibrillator; LBBB = left bundle branch block; LVT = left ventricular thickness, ECV = extracellular volume; LGE = late gadolinium enhancement.

**Table 2:** Demographic, clinical and cardiac MRI-phenotypic characteristics between HCM patients and rare disease patients.

Variable	**HCM** (n = 100)	Rare Disease Patients (n = 7)	*p*
Age(years)	43.1 ± 16.4	37.6 ± 16.5	0.403
Male gender, n (%)	64 (64)	2 (28.6)	0.062
BMI(kg/m^2^)	23.2 ± 3.7	19.5 ± 2.0	0.011 *
BSA(m^2^)	1.6 ± 0.2	1.5 ± 0.1	0.042 *
SBP(mmHg)	123.2 ± 16.9	115.0 ± 6.8	0.129
DBP(mmHg)	73.4 ± 10.2	72.8 ± 7.7	0.724
LVEF (%)	60.5 ± 9.2	43.8 ± 22.6	0.033 *
LVEDVi(mL/m^2^)	58.1 (50.8, 66.7)	71.7 (50.8, 95.0)	0.246
RVEF (%)	59.5 ± 10.3	50.0 ± 12.3	0.028 *
LVmassi (g/m^2^)	67.1 ± 33.3	127.8 ± 94.4	0.067
Max LVT(mm)	21.9 ± 6.0	22.1 ± 9.9	0.734
LGE (%)	7.8 (4.41, 14.56)	18.9 (14.05, 28.2)	0.003 *
Mapping			
Global native T1(ms)	1296.2 ± 66.6	1423.1 ± 93.3	*p* < 0.001 *
Global ECV (%)	29.9 ± 4.5	44.3 ± 11.5	*p* < 0.001 *
Basal native-T1(ms)	1291.2 ± 68.5	1413.7 ± 98.0	*p* < 0.001 *
Basal ECV (%)	29.6 ± 5.3	41.3 ± 15.2	0.013 *
Middle native-T1(ms)	1288.4 ± 75.8	1420.7 ± 80.7	*p* < 0.001 *
Middle ECV (%)	29.5 ± 5.3	43.8 ± 11.6	*p* < 0.001 *
Apex native-T1(ms)	1309.4 ± 81.5	1454.9 ± 133.7	0.008 *
Apex ECV (%)	30.2 ± 4.8	48.6 ± 8.9	*p* < 0.001 *

* *p* < 0.05; BMI = body mass index; SBP = systolic blood pressure; DBP = diastolic blood pressure; LVEF = left ventricular ejection fraction; LVmassi = LV mass index; Max LVT = LV maximal wall thickness; ECV = extracellular volume; LGE = late gadolinium enhancement. Continuous variables normally distributed are presented as mean ± SD, non-normally distributed as median and IQR.

## Data Availability

The data presented in this study are available on request from the corresponding author.

## Supplementary Materials

The following are available online at https://www.mdpi.com/article/10.3390/jcm10153339/s1. Table S1: List of the genes selected to perform the custom array.
