# Supplementary material for: The Value of Cardiac Magnetic Resonance Imaging in Identification of Rare Diseases Mimicking Hypertrophic Cardiomyopathy
Source: J Clin Med. 2021 Jul 28;10(15):3339. doi: 10.3390/jcm10153339 (PMC8348460; doi:10.3390/jcm10153339)
Supplement: Supplementary file 1 [file jcm-10-03339-s001.zip › jcm-1317037-supplementary.pdf]

Supplemental Files

The value of cardiovascular magnetic resonance imaging in identification of rare diseases mimicking hypertrophic cardiomyopathy

Table S1. List of the genes selected to perform the custom array.

Gene	mRNA	Chrs[*]	Segs(n)[#]	Seg（bp）[&]	Trans（n）[￥]
RRAGC	NM_001271851,NM_022157	chr1	8	3583	2
TMPO	NM_001032283,NM_001032284,NM_003276,NM_001307975	chr12	11	7993	4
RBM20	NM_001134363	chr10	15	8435	1
IDS	NM_001166550,NR_104128,NM_006123,NM_000202	chrx	11	7197	4
TGFB3	NM_001329938,NM_001329939,NM_003239	chr14	8	4308	3
IDUA	NR_110313,NM_000203	chr4	15	3418	2
KRAS	NM_004985,NM_033360	chr12	7	6689	2
HGD	NM_000187	chr3	15	3210	1
DOLK	NM_014908	chr9	2	2754	1
SYNE1	NM_182961,NM_001347702,NM_001347701,NM_033071	chr6	150	36288	4
CTNNA3	NM_013266,NM_001291133,NM_001127384	chr10	23	14469	3
SGSH	NM_000199	chr17	9	3668	1
EMD	NM_000117	chrx	7	2139	1
VCL	NM_003373,NM_014000	chr10	23	7086	2
MUT	NM_000255	chr6	14	5036	1
ANKRD1	NM_014391	chr10	10	2924	1
ALMS1	NM_015120	chr2	24	14572	1
LAMA4	NM_001105206,NM_001105208,NM_001105207,NM_001105209,NM_002290	chr6	40	10017	5
GUSB	NM_000181,NM_001293104,NM_001293105,NR_120531,NM_001284290	chr7	13	3399	5
NDUFS2	NM_004550,NM_001166159	chr1	16	3975	2
DTNA	NM_001198940,NM_001198941,NM_001198942,NM_001392,NM_001390,NM_001198939,NM_001391,NM_001198943,NM_001198944,NM_001198945,NM_032978,NM_032980,NM_001128175,NM_032975,NM_032979,NM_032981,NM_001198938	chr18	34	15055	17
DSG2	NM_001943	chr18	16	6903	1

CRYAB	NM_001330379,NM_001289808,NM_001289807,NM_001885	chr11	6	2283	4
FAH	NM_000137	chr15	15	2997	1
COX10	NM_001303	chr17	8	3852	1
DMD	NM_004010,NM_004006,NM_004014,NM_004016,NM_004013,NM_004021,NM_000109,NM_004017,NM_004018,NM_004020,NM_004019,NM_004015,NM_004011,NM_004022,NM_004012,NM_004023,NM_004009	chrx	92	23462	17
MYH6	NM_002471	chr14	40	8391	1
MYH7	NM_000257	chr14	41	8555	1
ILK	NM_001014794,NM_001014795,NM_001278442,NM_004517,NM_001278441	chr11	13	3388	5
DMPK	NM_001081560,NM_001288764,NM_001288765,NM_001288766,NM_001081563,NM_001081562,NM_004409	chr19	18	5955	7
NDUFV2	NM_021074	chr18	9	1830	1
AGL	NM_000644,NM_000646,NM_000642,NM_000028,NM_000643	chr1	38	11908	5
FUCA1	NM_000147	chr1	9	3017	1
PDLIM3	NM_001114107,NM_001257963,NR_047562,NM_001257962,NM_014476	chr4	10	3990	5
LMNA	NM_170708,NM_001282626,NM_001282624,NM_005572,NM_001257374,NM_170707,NM_001282625	chr1	20	6454	7
GLA	NM_000169	chrx	8	2268	1
ARSB	NM_198709,NM_000046	chr5	11	8125	2
TNNT2	NM_001001430,NM_001001431,NM_001001432,NM_001276347,NM_001276346,NM_000364,NM_001276345	chr1	20	2797	7
NEBL	NM_213569,NM_001173484,NM_006393	chr10	34	12526	3
RYR2	NM_001035	chr1	106	22115	1
PKP2	NM_001005242,NM_004572	chr12	15	5639	2
MYBPC3	NM_000256	chr11	36	6474	1
AGA	NM_001171988,NM_000027,NR_033655	chr4	10	3052	3
ACTN2	NM_001278344,NM_001103,NM_001278343	chr1	24	6760	3
MIB1	NM_020774	chr18	22	11117	1
TMEM43	NM_024334	chr3	13	4442	1
LDB3	NM_001080114,NM_001171611,NM_001080116,NM_001171610,NM_0010801	chr10	17	8041	6

	15,NM_007078				
MYPN	NR_045663,NM_001256268,NM_001256267,NM_032578,NR_045662	chr10	28	8749	5
CASQ2	NM_001232	chr1	12	3750	1
TMEM70	NR_033334,NM_017866,NM_001040613	chr8	5	2892	3
LAMA2	NM_001079823,NM_000426	chr6	67	13498	2
DSC2	NM_004949,NM_024422	chr18	18	6604	2
SCO2	NM_005138,NM_001169109,NM_001169110,NM_001169111	chr22	6	2855	4
PYGM	NM_001164716,NM_005609	chr11	21	5116	2
PLN	NM_002667	chr6	3	2314	1
TNNC1	NM_003280	chr3	7	1496	1
GNPTAB	NM_024312	chr12	22	7180	1
ACAT1	NM_000019	chr11	13	3234	1
GNS	NM_002076	chr12	15	6328	1
AGK	NM_018238	chr7	17	4225	1
PTPN11	NM_001330437,NM_002834,NM_080601	chr12	17	7877	3
NKX2-5	NM_001166176,NM_004387,NM_001166175	chr5	3	2561	3
NAGA	NM_000262	chr22	10	4676	1
MYOZ1	NM_021245	chr10	7	2383	1
PSEN1	NM_007318,NM_000021	chr14	13	7190	2
JUP	NM_002230,NM_021991	chr17	15	4708	2
NAGLU	NM_000263	chr17	7	3582	1
EYA4	NM_004100,NM_172105,NM_172103,NM_001301012,NM_001301013	chr6	21	7492	5
GYS1	NM_001161587,NM_002103,NR_027763	chr19	17	4918	3
SCN5A	NM_001099405,NM_001160160,NM_001160161,NM_198056,NM_001099404,NM_000335	chr3	30	10548	6
TTN	NM_001256850,NM_133379,NM_133437,NM_003319,NM_001267550,NM_133432,NM_133378	chr2	365	135554	7
FXN	NM_001161706,NM_181425,NM_000144	chr9	7	8254	3
RAF1	NM_002880	chr3	18	4625	1
NRAS	NM_002524	chr1	8	5304	1
ANO5	NM_213599,NM_001142649	chr11	23	8261	2
GALNS	NM_001323544,NM_001323543,NM_000512	chr16	15	3563	3
CALR3	NM_145046	chr19	10	2242	1

TCF21	NM_198392,NM_003206	chr6	4	4304	2
ABLIM1	NM_001003407,NM_001003408,NM_001322894,NM_001322882,NM_001322896,NM_001322897,NM_001322898,NM_001322899,NM_001322892,NM_001322883,NM_002313,NM_001322885,NM_001322893,NM_001322895,NM_006720,NM_001322886,NM_001322889,NM_001322884,NM_001322887,NM_001322891,NM_001322888,NM_001322890,NM_001322900	chr10	29	11111	23
HGSNAT	NM_152419	chr8	19	6614	1
SLC25A4	NM_001151	chr4	5	5120	1
POLG	NM_002693,NM_001126131	chr15	24	6098	2
RIT1	NM_001256821,NM_006912,NM_001256820	chr1	8	4818	3
COX15	NM_004376,NM_001320974,NM_078470,NM_001320976,NM_001320975	chr10	10	8639	5
MYL3	NM_000258	chr3	8	1779	1
GLB1	NM_001079811,NM_001135602,NM_001317040,NM_000404	chr3	18	4440	4
PSEN2	NM_012486,NM_000447	chr1	14	3448	2
DSP	NM_001008844,NM_004415,NM_001319034	chr6	25	11430	3
GBE1	NM_000158	chr3	17	4401	1
FHL1	NM_001330659,NM_001159700,NM_001159703,NM_001159704,NM_001167819,NM_001159699,NR_027621,NM_001449,NM_001159701,NM_001159702	chrx	16	7702	10
CAV3	NM_001234,NM_033337	chr3	3	2031	2
PRKAG2	NM_001040633,NM_001304527,NM_024429,NM_001304531,NM_016203	chr7	23	7756	5
CSRP3	NM_003476	chr11	8	2314	1
TPM1	NM_001018004,NM_001018006,NM_001018008,NM_001018020,NM_001330344,NM_001330346,NM_001330351,NM_000366,NM_001301289,NM_001301244,NM_001018007,NM_001018005	chr15	17	5099	12
HEXB	NM_000521,NM_001292004	chr5	17	4187	2
CTSA	NM_001127695,NM_001167594,NM_000308,NR_133656	chr20	16	3536	4
TNNI3	NM_000363	chr19	9	1754	1
ARSA	NM_001085425,NM_001085426,NM_001085428,NM_001085427,NM_000487	chr22	9	5215	5

MYL2	NM_000432	chr12	8	1679	1
BAG3	NM_004281	chr10	5	3269	1
LAMP2	NM_001122606,NM_002294,NM_013995	chrx	11	10388	3
TCAP	NM_003673	chr17	3	1563	1
GATAD1	NM_021167,NR_052016	chr7	6	5347	2
AGXT	NM_000030	chr2	12	2648	1
CTF1	NM_001330,NM_001142544	chr16	4	2314	2
GRHPR	NM_012203	chr9	10	2185	1
DNAJC19	NM_001190233,NR_033721,NM_145261,NR_033723,NR_033722,NR_046073	chr3	8	2746	6
ACTC1	NM_005159	chr15	8	4543	1
MYOZ2	NM_016599	chr4	7	3397	1
MYH7B	NM_020884	chr20	46	9334	1
DES	NM_001927	chr2	10	3198	1
NONO	NM_001145409,NM_001145408,NM_007363,NM_001145410	chrx	14	4368	4
TAZ	NM_181313,NM_000116,NM_181312,NR_024048,NM_181311,NM_001303465	chrx	12	3136	6
GAA	NM_000152,NR_134848,NM_001079803,NM_001079804	chr17	21	5275	4
SGCD	NM_000337,NM_001128209,NM_172244	chr5	10	11094	3
NEXN	NM_144573,NM_001172309	chr1	14	4539	2
ABCC9	NM_005691,NM_020297	chr12	40	10912	2

*chr: chromosome

#Segs (n): number of segments

&Segs (bp): length (bp) of the screened gene regions

¥Transc (n): Number of transcripts captured.
